# Virucidal Activity of Fogged Chlorine Dioxide- and Hydrogen Peroxide-Based Disinfectants against Human Norovirus and Its Surrogate, Feline Calicivirus, on Hard-to-Reach Surfaces

**DOI:** 10.3389/fmicb.2017.01031

**Published:** 2017-06-08

**Authors:** Naim Montazeri, Clyde Manuel, Eric Moorman, Janak R. Khatiwada, Leonard L. Williams, Lee-Ann Jaykus

**Affiliations:** ^1^Department of Food, Bioprocessing and Nutrition Sciences, North Carolina State University, RaleighNC, United States; ^2^Center for Excellence in Post-Harvest Technologies, North Carolina A&T State University, NC Research Campus, KannapolisNC, United States

**Keywords:** hydrogen peroxide, chlorine dioxide, fogged disinfectant, norovirus inactivation, surface disinfection, public health

## Abstract

Human norovirus (NoV) is the leading cause of foodborne illnesses in the United States. Norovirus is shed in high numbers in the feces and vomitous of infected individuals. Contact surfaces contaminated with bodily fluids harboring infectious virus particles serve as vehicles for pathogen transmission. Environmental stability of NoV and its resistance to many conventional disinfectants necessitate effective inactivation strategies to control the spread of virus. We investigated the efficacy of two commercial disinfectants, hydrogen peroxide (7.5%) and a chlorine dioxide (0.2%)-surfactant-based product using a fogging delivery system against human NoV GI.6 and GII.4 Sydney strains as well as the cultivable surrogate, feline calicivirus (FCV) dried on stainless steel coupons. Log_10_ reductions in human NoV and FCV were calculated utilizing RNase RT-qPCR and infectivity (plaque) assay, respectively. An improved antiviral activity of hydrogen peroxide as a function of disinfectant formulation concentration in the atmosphere was observed against both GII.4 and FCV. At 12.4 ml/m^3^, hydrogen peroxide achieved a respective 2.5 ± 0.1 and 2.7 ± 0.3 log_10_ reduction in GI.6 and GII.4 NoV genome copies, and a 4.3 ± 0.1 log_10_ reduction in infectious FCV within 5 min. At the same disinfectant formulation concentration, chlorine dioxide-surfactant-based product resulted in a respective 1.7 ± 0.2, 0.6 ± 0.0, and 2.4 ± 0.2 log_10_ reduction in GI.6, GII.4, and FCV within 10 min; however, increasing the disinfectant formulation concentration to 15.9 ml/m^3^ negatively impacted its efficacy. Fogging uniformly delivered the disinfectants throughout the room, and effectively decontaminated viruses on hard-to-reach surfaces. Hydrogen peroxide delivered by fog showed promising virucidal activity against FCV by meeting the United States EPA 4-log_10_ reduction criteria for an anti-noroviral disinfectant; however, fogged chlorine dioxide-surfactant-based product did not achieve a 4-log_10_ inactivation. Future investigation aimed at optimizing decontamination practices is warranted.

## Introduction

Human norovirus (NoV) is the leading etiologic agent of acute gastroenteritis, accounting for 48% of all foodborne outbreaks in the United States (Hall et al., 2014). Human NoV is a non-enveloped virus with a positive-sense RNA genome belonging to the family *Caliciviridae* (Green, 2007). The virus is transmitted either directly through fecal-oral or vomit-oral routes, or indirectly through contact with contaminated surfaces, or through the consumption of contaminated food and water. Once deposited on surfaces, human NoV can remain infectious for several weeks (Escudero et al., 2012; Lopman et al., 2012; Hall et al., 2014). Environmental stability of human NoV is enhanced by resistance to commercial sanitizers and disinfectants, including alcohol-based hand sanitizers and hypochlorite at regulated concentrations (Liu et al., 2010; Tung et al., 2013; Cromeans et al., 2014; Cook et al., 2016). These unique traits of human NoV contribute to the high number of outbreaks observed annually in close quarter environments such as cruise ships, long-term care facilities, and schools, as well as in association with food service (Lopman et al., 2012; Cook et al., 2016). Therefore, innovative methods for inactivation of NoV from these environments where frequent human contact with surfaces is expected are needed to control the spread of the pathogen.

Conventional methods for disinfection of contaminated surfaces are often time-consuming and labor-intensive. Additionally, manual disinfection of surfaces relies on operator compliance to achieve an optimal efficacy. Considering these shortcomings, automated disinfection methods have become increasingly popular. Chlorine dioxide (ClO_2_) and hydrogen peroxide (H_2_O_2_) are two strong oxidizing agents with a broad antimicrobial activity offering a promising potential as contact surface sanitizers (Hoehn et al., 2010; Tuladhar et al., 2012). Gaseous delivery of these disinfectants has shown superior antimicrobial activity over aqueous forms by being more diffusible, penetrable and able to access areas beyond the reach of liquid sanitizers and hard-to-clean sites (Morino et al., 2011; Tuladhar et al., 2012; Yeap et al., 2015).

Despite the widespread use of ClO_2_ and H_2_O_2_ as surface disinfectants, to the best of our knowledge, there is no study in the literature that characterizes the efficacy of these disinfectants against human NoV using a fogging system. We sought to characterize the antiviral activity of two commercially available ClO_2_- and H_2_O_2_-based disinfectants when delivered by a portable fogging device against two epidemiologically important human NoV outbreak strains GI.6 and GII.4 as well as the frequently used cultivable surrogate feline calicivirus (FCV) on stainless steel coupons. In the absence of a practical human NoV cell culture system, we utilized real-time polymerase chain reaction (RT-qPCR) preceded by RNase treatment for the detection and quantification of intact, presumptively infectious virus particles (Knight et al., 2013; Manuel et al., 2015). A standard plaque assay technique was used to determine reduction in infectious titer of FCV particles following exposure to the disinfectants. The experiments for each disinfectant were performed separately with no intention of being a comparative study, although the results of each are described here. This research provides evidence of the efficacy of an antiviral disinfectant delivery system for inactivation of human NoV in enclosed areas.

## Materials and Methods

### Disinfectants

Samples of the two commercial products used in this study were kindly provided courtesy of M. Quinoy (AeroClave^TM^, Winter Park, FL, United States). The disinfectants were (i) H_2_O_2_ 7.5% (inert ingredients 92.5%, United States EPA registration No. 83046-1, AeroClave); and (ii) Vital Oxide^®^ (United States EPA registration No. 82972-1, Vital Solutions, West Palm Beach, FL, United States), a ClO_2_-surfactant-based product with United States EPA approval of anti-noroviral efficacy based on infectivity assay against the cultivable surrogate FCV (United States Environmental Protection Agency, 2016b), and claims active ingredients as 0.20% ClO_2_, 0.125% alkyl (60% C14, 30% C16, 5% C12, 5% C18) dimethyl benzyl ammonium chloride, 0.125% alkyl (68% C12, 32% C14) dimethyl ethylbenzyl ammonium chloride, and 99.55% as inactive ingredients.

### Preparation of Virus Stocks

Fecal specimens collected from outbreaks, and confirmed positive for human NoV GI.6 and GII.4 Sydney strains by sequencing, were kindly provided by S. R. Greene (North Carolina Division of Public Health, Raleigh, NC, United States). Prior to use in experiments, we reconfirmed their identity using genogroup-specific RT-qPCR, as explained below. A 20% suspension (w/v) was prepared in phosphate-buffered saline solution (PBS; pH 7.2), clarified by centrifugation (3,100 × g for 2 min at room temperature), and stored at -80°C until use.

Feline calicivirus strain F9 (FCV) was propagated in Crandell Rees feline kidney (CRFK) cells as previously described (Tung et al., 2013). Briefly, preparation of virus stocks was done by infecting a 90% confluent CRFK monolayer at a multiplicity of infection of 0.6. The cells were incubated at 37°C with 5% CO_2_ until >90% of cells displayed cytopathic effects. The cells were lysed by three freeze-thaw cycles at -80°C to release viral particles. Lysates were clarified by centrifugation, passed through a 0.2-μm filter, aliquoted and stored at -80°C until use.

### Coupon Preparation and Inoculation

Non-adhesive stainless steel embossing tape (DYMO Co, Berkeley, CA, United States) were utilized as carriers. The tape was cut into 2.5 cm × 5.0 cm pieces, degreased in acetone, and sterilized by autoclaving for 15 min at 121°C. Each coupon was inoculated by placing a 25-μl aliquot of virus stock at the center of each strip (6–7 log_10_ titer), air-dried in a biosafety hood for 45 min, and immediately used for each experiment. All procedures were carried out in accordance to the United States EPA confirmatory virucidal effectiveness test (United States Environmental Protection Agency, 2016a).

### Experimental Conditions and Virus Treatments

**FIGURE 1:**
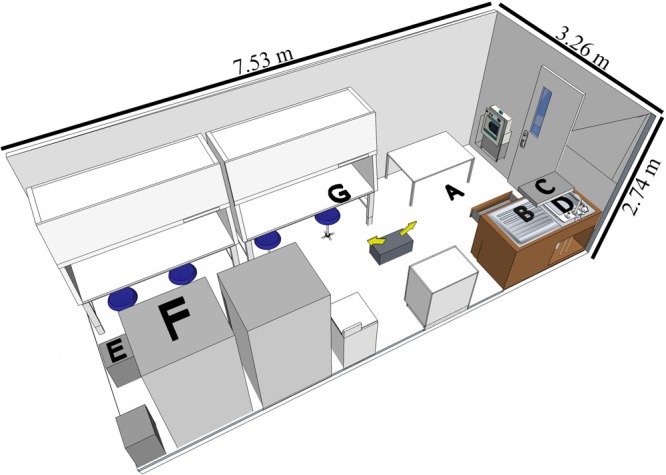
Diagram of BSL-3 laboratory setup with the coupon locations indicated. The fog generator was placed on the ground as indicated by the two arrows showing the directions of fogging. Graphical representation of the laboratory is to scale (room layout created by SketchUp Make 2016, Trimble Navigation Ltd.). Coupon locations as follows: (A) (floor) for both experiments; (B) (counter) and (C) (shelf) for H_2_O_2_-based product; (D) (sink), (E) (drawer), (F) (top of the refrigerator) and (G) (inside the hood while the door open) for the ClO_2_-based disinfectant.

A BSL-3 laboratory (7.53 L × 3.26 W × 2.74 H meters, **Figure 1**) in North Carolina Research Campus (Kannapolis, NC, United States) was used for the study. The disinfectants were tested in separate experiments with different assigned locations of the coupons (**Figure 1**) representative of contamination spots on easy-to-reach and hard-to-reach areas for cleaning purposes.

After coupon placement, the room was exited, the air handler unit was turned off, and the fogged disinfectant was delivered into the room (relative humidity 60%, 21°C) using the automated Room Decontamination System 3110 (AeroClave), located at the center of the laboratory floor. The machine was equipped with two nozzles, that according to the manufacturer, was capable of generating fogs at 30 ml/min per nozzle at opposite directions. This allowed us to adjust fog generation time, until desired disinfectant formulation concentrations of 7.1–15.9 ml/m^3^ were achieved. The target disinfectant formulation concentration was held for 5 min for H_2_O_2_ and 10 min for ClO_2_-surfactant-based product (according to the manufacturer recommendations), then the ventilation was resumed, and aeration was allowed for 20 min to remove the fogged disinfectant. Exposed carriers were immediately suspended in 3–5 ml PBS in a 15-ml conical tube and vortexed vigorously for 30 s to facilitate virus elution. Eluted viruses were aliquoted and stored frozen at -80°C prior to analysis. For each replicate, positive controls were prepared by placing the inoculated coupons on the bench just outside the BSL-3 laboratory entrance room for the duration of each experiment so that they were not exposed to the disinfectants. Virus log_10_ inactivation was calculated by subtracting the titer of the disinfectant-treated inoculated coupons from the titer of positive controls.

### Human Norovirus RT-qPCR Analysis

Prior to analysis by RT-qPCR, human NoV GI.6 and GII.4 inoculated samples were subjected to an RNase treatment, as previously reported (Manuel et al., 2015). Pretreatment of eluted viral particles with RNase serves as an alternative method to discriminate between infectious and non-infectious viruses through degradation of free floating viral RNA or exposed RNA from partially destructed capsids, preventing them from being amplified during RT-qPCR (Topping et al., 2009; Knight et al., 2013). Briefly, 100-μl of eluate was mixed with 1 U of RNase ONE^TM^ ribonuclease and 1 × reaction buffer (Promega, Madison, WI, United States), and incubated at 37°C for 15 min. Samples were then placed on ice for 5 min to stop the reaction. The viral RNA was immediately extracted with an automated NucliSENS^®^ easyMag^®^ system (BioMérieux, St. Louis, MO, United States) per manufacturer’s instructions, eluted in a proprietary buffer, and stored at -80°C until RT-qPCR analysis.

In all experiments, RT-qPCR amplification targeted the ORF1-ORF2 junction of the human norovirus genome using COG1F/COG1R primers and Ring1(a)/Ring1(b) probes for GI.6 (Kageyama et al., 2003), and JJV2F/COG2R primers and Ring2 probe for GII.4 (Jothikumar et al., 2005). Estimation of genomic copies was performed by comparison with a calibration curve established using RNA transcripts of the ORF1-ORF2 junction of the human norovirus genome (Escudero et al., 2012). The log_10_-transformed RNA genomic copies were plotted against the threshold cycle (C_t_) value (threshold 30) using linear regression to make the calibration curve. Negative amplification control (water) and positive amplification controls (diluted GI and GII RNA transcripts) were incorporated in each RT-qPCR run. All RT-qPCR analyses were performed on a Bio-Rad CFX96 Touch^TM^ Real-Time PCR Detection System (Hercules, CA, United States).

RT-qPCR conditions varied slightly depending on the experiment. For the H_2_O_2_ experiment, a One-step iScript^TM^ RT Supermix kit (Bio-Rad, Hercules CA, United States) was used in 25-μl master mix composed of 2.5 μl of viral RNA, 200 nM of primers, 200 nM of fluorescently labeled TaqMan probe, 1 × Bio-Rad PCR reaction buffer (Bio-Rad), and 0.5 μl Bio-Rad iScript RT mix. The reaction mixture was subjected to a one-step thermal cycling profile under the following amplification conditions: (i) reverse transcription for 10 min at 50°C, (ii) initial denaturation for 5 min at 95°C, and (iii) 45 cycles of 15 s at 95°C and 30 s at 55°C.

For the experiments on ClO_2_-surfactant-based product, a SuperScript^TM^ III One-Step RT-PCR with Platinum^®^ Taq High Fidelity DNA polymerase (Invitrogen, Carlsbad, CA, United States) was used. The reaction volume of 25 μl was composed of 2.5 μl of RNA template, 1 × reaction mix, 0.5 μl SuperScript^®^ III RT/Platinum^®^ Taq mix, 0.25 U RNasin^®^ Plus ribonuclease inhibitor (Promega), 200 nM of each primer and 200 nM of GII probe or 120 nM of each of the two GI probes. The RNA was reverse-transcribed at 50°C for 15 min, the Platinum Taq polymerase was activated at 95°C for 2 min, then followed by thermal cycling for 15 s at 95°C, 30 s at 54°C, and 30 s at 72°C for a total of 45 cycles.

### Feline Calicivirus (FCV) Infectivity Assay

Infectious titers of FCV were determined using the United States EPA standard plaque assay technique (United States Environmental Protection Agency, 2016a). Briefly, CRFK cell monolayers at 80–90% confluency were infected with 450 μl of 10-fold serially diluted eluates. After overlay and incubation for 2–3 days at 37°C in 5% CO_2_, cells were fixed in 3.7% formaldehyde, and plaques visualized by staining with 0.1% (w/v) crystal violet solution. The cells were rinsed with water and plates with 5–50 plaque-forming units (PFU) were used to determine infectious virus titer. Neutralizer control (using PBS) and cell viability controls were included in accordance with the United States EPA method (United States Environmental Protection Agency, 2016a).

### Statistical Analysis

Each experiment tested a single disinfectant formulation concentration, and was replicated three times with duplicate measurements. All data are reported as mean ± standard error. Statistical analysis was done by one-way ANOVA followed by Tukey’s HSD for pair-wise comparisons of means using RStudio (Version 0.99.903, RStudio Inc., Boston, MA, nited States). A *p*-value of smaller than 0.05 was considered statistically significant.

## Results

The purpose of this research was to evaluate the applicability of both H_2_O_2_ (7.5%) and ClO_2_ (0.2%)-surfactant-based disinfectants against human NoV and the cultivable surrogate virus, FCV, on stainless steel contact surfaces. We employed RNase RT-qPCR on human NoV to select for intact virus particles, and plaque assay to assess infectivity of FCV. Highest quantifiable degree of virus inactivation, based on virus stock concentration and assay detection limits, was 2.1–2.8 and 3.2–3.7 log_10_ genomic copies for NoV GI.6 and GII.4 Sydney, respectively, and 4.2–5.1 log_10_ PFU for FCV.

### Hydrogen Peroxide (H_2_O_2_)

**FIGURE 2:**
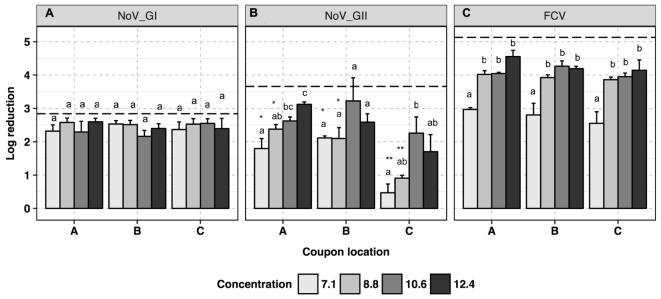
Efficacy of H_2_O_2_ fogging on GI.6 and GII.4 Sydney human NoV and feline calicivirus (FCV) at various disinfectant formulation concentrations (ml/m^3^). For each location within each section (virus strain): (1) uppercase letters denote coupons location as shown in **Figure 1**; (2) lowercase letters indicate statistically significant differences in each location across disinfectant formulation concentrations; (3) asterisks denote significant differences for each disinfectant formulation concentration across locations (only observed for human NoV GII.4 on the samples located on location C, shelf). Results are expressed as log_10_ reduction in genomic copies by RNase RT-qPCR for human NoV, and plaque-forming units (PFU) for FCV. Error bars represent standard error of the mean. All experiments were performed in triplicate. Long-dashed lines represent the highest quantifiable degree of virus log_10_ inactivation.

No trend was observed for human NoV GI.6 reduction as a function of H_2_O_2_-based disinfectant formulation concentration (*p* > 0.05, **Figure 2A** and Supplementary Data Sheet 1). However, increasing the concentration from 7.1 to 12.4 ml/m^3^ enhanced viral genomic copy number reduction for GII.4 from 1.5 ± 0.3 to 2.7 ± 0.3 log_10_ copies, respectively (*p* < 0.05, **Figure 2B**). For GII.4 samples, the inoculated coupons placed on the shelf (site C, **Figure 1B**) and exposed to 7.1 and 8.8 ml/m^3^ H_2_O_2_ had significantly lower overall reductions (0.7 log_10_) as compared to the coupons placed on a counter top (site B, 2.0 log_10_, *p* < 0.05) or floor (site A, 2.2 log_10_, *p* < 0.05). No significant location effect for carriers was observed for GI.6 samples (*p* > 0.05). Norovirus GII.4 displayed a significantly lower reduction in RNA genomic copies as compared to GI.6 when carriers were placed on either the shelf or counter and exposed to lower disinfectant formulation concentrations of H_2_O_2_ (*p* < 0.05, **Figures 2A,B**).

Virucidal activity of H_2_O_2_ against FCV was enhanced as the disinfectant formulation concentration of fogged disinfectant increased in the atmosphere, reaching the maximum reduction of 4.3 log_10_ in infectious FCV particles (*p* < 0.05, **Figure 2C**). At all locations, at least 10.6 ml/m^3^ H_2_O_2_ was required to ensure a 4-log_10_ reduction for infectious FCV. No location effect was observed for FCV inactivation in any of the samples tested at any disinfectant formulation concentration (*p* > 0.05).

### ClO_2_-Surfactant-Based Product

**FIGURE 3:**
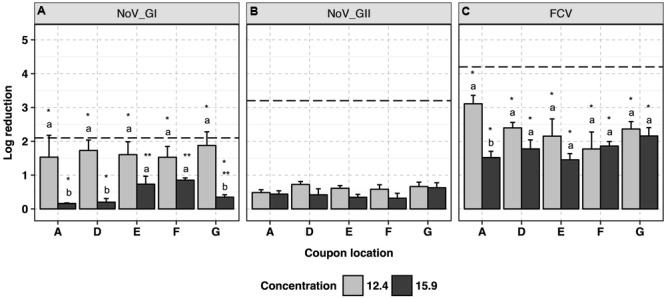
Efficacy of ClO_2_-surfactant-based product fogging on GI.6 and GII.4 Sydney human NoV and FCV at various disinfectant formulation concentrations (ml/m^3^). For each virus: (1) uppercase letters denote coupons location as shown in **Figure 1**; (2) lowercase letters indicate statistically significant differences in each location across disinfectant formulation concentrations; (3) asterisks denote significant differences for each concentration across locations [statistical analysis was not carried out for human NoV GII Sydney due to the negligible reduction (<1 log_10_) observed in those samples]. Results are expressed as log_10_ reduction in genomic copies by RNase RT-qPCR for human NoV, and PFU for FCV. Error bars represent standard error of the mean. All experiments were performed in triplicate. Long-dashed lines represent the highest quantifiable degree of virus log_10_ inactivation.

This product was used at the recommended disinfectant formulation concentration of 12.4 ml/m^3^ and a higher concentration of 15.9 ml/m^3^ to assess any enhanced virucidal activity. Human NoV GI.6 and GII.4 strains showed 1.7 ± 0.2 and 0.6 ± 0.1 log_10_ reductions in genome copy number, respectively, following application at 12.4 ml/m^3^ (**Figures 3A,B** and Supplementary Data Sheet 1) with no impact of coupon location on the efficacy of the disinfectant (*p* > 0.05). The antiviral efficacy decreased (*p* < 0.05) when the product was applied at the higher disinfectant formulation concentration (15.9 ml/m^3^), wherein less than 1.0 log_10_ reduction in genome copies was achieved in the majority of the samples; this was consistent across the replicates. At 15.9 ml/m^3^, marginal differences in GI.6 log_10_ inactivation were observed as a function of the location of the coupons, and were not always statistically significant (*p* > 0.05).

The average log_10_ reduction in infectious FCV titer as the result of exposure to ClO_2_-surfactant-based product was 2.4 ± 0.2 at 12.4 ml/m^3^ versus 1.8 ± 0.1 at 15.9 ml/m^3^ (**Figure 3C**). Log_10_ reduction in infectious FCV was, respectively, 0.7 and 1.7 log_10_ higher than GI.6 and GII.4 log_10_ genome copy number reductions when 12.4 ml/m^3^ was applied (*p* < 0.05). Similar to the data for human NoV, the product was uniformly efficacious against FCV across coupon locations with a reduced efficacy at higher disinfectant formulation concentration. Slight condensation on the ground around the fog generator were observed when ClO_2_-surfactant-based product was applied at 15.9 ml/m^3^.

## Discussion

Fomites (porous and non-porous surfaces) can be contaminated with human NoV through direct contact with the feces or vomitus shed by infected individuals, and serve as vehicles to spread the virus. Findings have demonstrated the stability of human NoV on hard surfaces and their resistance to common disinfectants, contributing to the NoV persistence in the environment and rapid transmissibility (Escudero et al., 2012; Tung et al., 2013; Liu et al., 2015; Pringle et al., 2015).

We investigated the antiviral activity of two disinfectants against human NoV GI.6 and GII.4 and FCV, the cultivable surrogate, using a fogging device. Human NoV GII.4 fecal suspensions dried on stainless steel coupon have been shown to be stable for at least 240 min when exposed to air under standard laboratory conditions (Manuel et al., 2015). Therefore, the observed virus reduction in our experiment could be representative of virus inactivation as the result of the exposure to the disinfectants. Our experimental design includes four unique features: (i) incorporation of an RNase treatment prior to RT-qPCR for human NoV to provide data more representative of viral infectivity by targeting intact viruses (Knight et al., 2013); (ii) use of two different human NoV genogroups to evaluate potential genotype-associated differences in response to the disinfection; (iii) use of a cultivable surrogate to evaluate virus infectivity in parallel, and as recommended by the United States EPA for registration of anti-norovirus product claims (United States Environmental Protection Agency, 2016a); and (iv) consideration of the impact of contamination location on fogging efficacy. The experiments on each disinfectant were performed separately and this is not intended to be a comparative study. In addition, it should be noted that the virucidal performance observed here is a function of the method of application, formulation concentration in the atmosphere and the contact times under which this study was conducted.

In general, both disinfectants were effective against human NoV and FCV. We observed a higher reduction in human NoV GI.6 viral genomic copies at low formulation concentrations of the disinfectants when compared to GII.4 located in the same area (**Figures 2, 3**). This suggests that GII.4 could be more resistant than GI.6 to the disinfectants, in agreement with a previous report of human NoV susceptibility to alcohols, chlorine, and high hydrostatic pressure (Cromeans et al., 2014). Given that the majority of human illnesses are caused by GII.4 epidemic strains (Pringle et al., 2015), it is tempting to link the enhanced environmental resistance of these strains to their widespread prevalence, although additional studies are needed to test this hypothesis. Although not the focus of our research, slight variation in the resistance of human NoV strains of a certain genogroup to disinfectants may exist, as a recent study reported a lower resistance of GII.4 Sydney to alcohol as compared with GII.4 New Orleans strain (Park et al., 2016).

To our knowledge, this study represents the only one of its kind to investigate the impact of fogged H_2_O_2_ and a ClO_2_-surfactant-based product on both outbreak-associated human NoV strains, GI.6 and GII.4, and the cultivable surrogate FCV. We found that fogged H_2_O_2_ could inactivate GI.6 and GII.4 human NoV by 2.4 and 1.4 log_10_ at the lowest applied disinfectant formulation concentration (7.1 ml/m^3^). In a similar study, Tuladhar et al. (2012) examined the inactivation of human NoV GII.4 positive stool under exposure to fogged H_2_O_2_ and observed less than 1.0 log_10_ reduction in genomic copies; however, the authors did not perform RNase treatment prior the RT-qPCR and hence their results may underestimate reduction in viral genomic copies and product efficacy.

In the case of ClO_2_-surfactant-based product, using monoclonal antibody-conjugated immunomagnetic beads to select for infectious viruses, Liu et al. (2015) demonstrated no reduction for human NoV GI and GII in suspension assays. Even though a superior antiviral activity for chemical disinfectants is usually expected when antiviral activity is examined in suspension as compared with surface assays (Morino et al., 2011), we found that ClO_2_-surfactant-based product, when delivered as a fog at 12.4 ml/m^3^ provided 1.7 and 0.6 log_10_ reduction in GI.6 and GII.4 Sydney genomic copies when inocula were dried on stainless steel coupons. As stated above, the differences in these results may be a function of methodological approaches to estimating surviving infectious human NoV.

Both H_2_O_2_ and ClO_2_ are strong oxidizing agents that can destroy both proteins and nucleic acids (McDonnell and Russell, 1999; Yeap et al., 2015). Given the structure of enteric viruses, it would be logical to assume that the initial hit to the virus occurs at the capsid, after the destruction of which follows attack of viral RNA. Our data supports the notion that both disinfectants attack the virus capsid; however, given our experimental design, no conclusion on the impact of the disinfectants on viral genomes could be drawn. A recent study published by our group revealed that copper appears to inactivate human NoV by attacking both viral genome and capsid (Manuel et al., 2015). Since the antimicrobial mechanism of action of copper is similar to that of H_2_O_2_ (i.e., generation of reactive free radicals), it is reasonable to speculate that H_2_O_2_ fogging is likely to also impact the viral genome of human NoV, although this requires further confirmatory studies. Gaseous ClO_2_ has been shown to inactivate murine norovirus, a cultivable human NoV surrogate, through degradation of both viral capsid protein and genome (Yeap et al., 2015). In the case of sodium hypochlorite, however, no significant degradation of the viral genome at 1,000 ppm was observed for human NoV GI, GII and the surrogates, except for FCV (Cromeans et al., 2014). Surface assays showed that sodium hypochlorite, even at high concentrations (e.g., 5,000 ppm), does not seem to have a significant role in degradation of the viral genome after 4 min contact with a NoV GII.4 fecal suspension inoculated on steel coupons (Park and Sobsey, 2011).

Evaluating the virucidal efficacy of a disinfectant solely based upon reduction in viral genomic reduction may underestimate the efficacy of disinfectants (Yeargin et al., 2015); therefore, testing the infectious titer of a non-enveloped cultivable surrogate provides further evidence on the antiviral activity of a disinfectant (Tung et al., 2013; Cromeans et al., 2014). Among the human NoV cultivable surrogates, FCV is the approved surrogate established by the United States EPA to assess the anti-noroviral efficacy of disinfectants (United States Environmental Protection Agency, 2016a). The 4.3 log_10_ reduction in infectious titer we reported for FCV during exposure to H_2_O_2_ fog is in accordance with a previous study (Bentley et al., 2012). After exposure to fogged ClO_2_-surfactant-based product, we observed a comparatively lower reduction of FCV titer (2.4 log_10_) at 12.4 ml/m^3^. The efficacy of disinfection depends on a number of factors including mode of application, disinfectant concentration, contact time, organic load of the inoculum, and virus type. Since there have not been previous fogging studies with FCV exposed to chlorine dioxide by fog, the only comparisons that can be made are hard surface studies with ClO_2_ gas. Morino et al. (2011) demonstrated less than a 3 log_10_ reduction in FCV titer even after a 4-h contact time with 0.05 ppm ClO_2_ gas. Other surrogates such as murine norovirus are likely to be more resistant than FCV to oxidizing disinfectants such as sodium hypochlorite (Cromeans et al., 2014), meaning this disinfectant may be even less efficacious than reported in our study.

To test the impact of contamination location on the efficacy of the disinfection system, we placed the coupons at different elevations and distances to the fogger (**Figure 1**). The only location effect occurred in H_2_O_2_ experiment when carriers containing GII.4 Sydney virus were placed on the shelf (site C, 1.6 m height) and subjected to the two lowest disinfectant formulation concentrations of 7.1 and 8.8 ml/m^3^ (**Figures 1, 2B**). Interestingly, a previous study failed to observe any location effect when carriers containing poliovirus were placed on top of a 2.0 m high closet and subjected to H_2_O_2_ fogging using a similar delivery mechanism and fogging parameters as compared to our study (Tuladhar et al., 2012). In the case of ClO_2_-surfactant-based product, no location effect was observed for any of the viruses, even when the product was fogged at the recommended disinfectant formulation concentration of 12.4 ml/m^3^.

When used at a disinfectant formulation concentration of 15.9 ml/m^3^, the efficacy of fogged ClO_2_-surfactant-based product achieved a 0.4 log_10_ reduction in both GI and GII and 1.8 log_10_ reduction in FCV. The negative impact of increased ClO_2_-surfactant-based product concentration on virus inactivation was observed both in human NoV RNA genome copies as well as FCV PFU across replicates. Though not explored experimentally, we believe that over-saturation of the fog at disinfectant formulation concentrations higher than that recommended (12.4 ml/m^3^) resulted in condensation of the disinfectant and more rapid removal from the atmosphere. In a similar study, a 3.0 log_10_ reduction in murine norovirus was reported after 2 min exposure to 2.5 ppm ClO_2_ gas; and when 2.0 ppm ClO_2_ gas was applied, a similar virus inactivation could be obtained only after extending the contact time to 5 min (Yeap et al., 2015). Therefore, we speculated that the efficacy of the ClO_2_-surfactant-based product tested in this study might be enhanced if applied at the recommended disinfectant formulation concentration of 12.4 ml/m^3^ but for a longer contact time.

Our observation of adequate dispersion of both products when used at appropriate disinfectant formulation concentrations illustrates a significant advantage to fogging in that it can uniformly provide exposure to disinfectant throughout the room, covering even hard-to-reach surfaces. Further, since fogging is semi-automated, covers a wide area, and should inactivate airborne virus (not evaluated in our study), it should allow higher sanitation efficacy, increased compliance, and better worker productivity. A disadvantage of these systems is their high cost relative to conventional surface sanitation methods and that they can only be used in semi-enclosed spaces which must be evacuated for 30–60 min before reentry. They are also designed to be used on pre-cleaned surfaces. While we did not perform experiments using additional simulated organic (soil) load, previous studies using oxidizing disinfectants including H_2_O_2_, ClO_2_ or sodium hypochlorite against a variety of human norovirus surrogates (murine norovirus and FCV) have shown that organic load significantly reduces virucidal efficacy (Urakami et al., 2007; Ayyildiz et al., 2009; Morino et al., 2011). Despite the reasonable virucidal efficacy achieved by applying the 7.5% H_2_O_2_ disinfectant by fogging, corrosivity concerns after long-term use remain (Rutala et al., 2008). Unfortunately, at lower concentrations H_2_O_2_ may lose its anti-noroviral activity (Kingsley et al., 2014).

Overall, this study represents the only one of its kind to investigate the impact of fogging on multiple human NoV strains (GI.6 and GII.4) along with the cultivable surrogate FCV under the same set of experimental conditions. Major findings from this study include evidence that exposure to H_2_O_2_ fogging results in an approximate 2.5 and 2.7 log_10_ reduction in RNA genomic copies for human norovirus GI.6 and GII.4 Sydney (respectively), and a 4.3 log_10_ reduction in infectious FCV at 12.4 ml/m^3^. On the other hand, the ClO_2_-surfactant-based product was mildly efficacious against the tested viruses, achieved only a 1.7 and 0.6 log_10_ reduction in RNA genomic copies for human NoV GI.6 and GII.4 (respectively), and 2.4 log_10_ reduction in PFU for FCV when used at the recommended disinfectant formulation concentration of 12.4 ml/m^3^. Both experiments indicated that GII strains of human norovirus may be more resistant to the fogged disinfectants than GI strains. In conclusion, fogging systems, especially when used with hydrogen peroxide disinfectant, show promise application for NoV disinfection of enclosed areas, allowing the disinfectant to saturate the air and reach hard-to-disinfect surfaces. It is important to note that due to differences in experimental design and methods, this work does not allow for the direct comparison of the efficacy of the tested disinfectants delivered as a fog. Future studies should focus on improving the surface decontamination by optimizing the application technologies and disinfection parameters.

## Author Contributions

NM, CM, and EM designed and performed the experiments, and contributed to the preparation of the manuscript. JK and LW provided the BL-3 facility and technical insights on utilization of facility. L-AJ led the research.

## Conflict of Interest Statement

The authors declare that the research was conducted in the absence of any commercial or financial relationships that could be construed as a potential conflict of interest.
